# Exploratory investigation of the outcomes of wheelchair provision through two service models in Indonesia

**DOI:** 10.1371/journal.pone.0228428

**Published:** 2021-06-01

**Authors:** Megan E. D’Innocenzo, Jonathan L. Pearlman, Yasmin Garcia-Mendez, Stephanie Vasquez-Gabela, Christina Zigler, Perth Rosen, Eviana Hapsari Dewi, Ignatius Praptoraharjo, Anand Mhatre

**Affiliations:** 1 Department of Rehabilitation Science and Technology, University of Pittsburgh, Pittsburgh, Pennsylvania, United States of America; 2 Department of Population Health Science, Duke University, Durham, North Carolina, United States of America; 3 United Cerebral Palsy (UCP) Wheels for Humanity, Chatsworth, California, United States of American; 4 Department of Health Policy and Management, Gadjah Mada University, Yogyakarta, Indonesia; Università degli Studi di Perugia, ITALY

## Abstract

The World Health Organization (WHO) estimates that only 17–37% of the approximately 77 million people who need a wheelchair have access to one. Many organizations are trying to address this need through varying service delivery approaches. For instance, some adhere to WHO’s recommended 8-steps service approach while others provide wheelchairs with little to no service. There is limited and sometimes conflicting evidence of the impact of the WHO’s recommendations on the outcomes of wheelchair provision. To help build this evidence, we \explored outcomes of two groups of users who received their wheelchairs through two service models over time. The 8-Steps group (*n* = 118) received a wheelchair selected from a range of models from service providers trained using the WHO process, and the standard of care (SOC) group (*n* = 24) received hospital-style wheelchairs and without clinical service. Interviews were conducted at baseline and at follow-up 3 to 6 months after provision, to collect data about wheelchair usage, satisfaction, skills, maintenance and repairs, and life satisfaction. Across-group statistical comparisons were not appropriate due to significant differences between groups. In general, participants used their wheelchairs every day but reported very low mobility levels (<500 meters for the 8-steps group, and <100 meters for the SOC group.) The 8-steps group used their wheelchair for either between 1–3 hours per day, or more than 8 hours per day. The SOC used it between 1 and 3 hours per day. Overall, wheelchair usage and wheelchair skills decreased over the 3- to 6-month data collection timeline. Wheelchair breakdowns were common in both groups emphasizing the need for maintenance, occurring more frequently in the 8-Steps (28.8%) compared to the SOC group (8%), and emphasizing the need for maintenance services. No significant differences were found when comparing device satisfaction across wheelchairs types. Our results emphasize the need for routine maintenance to address frequent wheelchair breakdowns. Our results also demonstrate a large disparity in several outcome variables across groups which motivates future studies where across-group comparisons are possible.

## Introduction

There is a significant unmet need for appropriate wheelchairs around the world. Using population-based estimates published by WHO, approximately 77 million people worldwide currently require the use of a wheelchair for mobility [1]. Data collected in several less-resourced settings (LRS) on access to assistive technologies suggests that only between 17% and 37% have access to appropriate assistive technologies, such as wheelchairs. Based on these data, an estimated 33–65 million people who need wheelchairs do not have access to them. This large unmet need has motivated governments, private companies, and not-for-profit organizations to provide wheelchairs through a range of largely uncoordinated service provision and supply chain approaches for the past several decades [2, 3]. Concerns that some of these approaches lacked the desired impact (e.g. [4, 5]) motivated a multi-year effort to establish standards related to service and product quality. A consensus conference held in 2006 led by the WHO [6] resulted in the development and publication of consensus guidelines [7] on manual wheelchair provision, and a set of consensus-based training packages to educate wheelchair service providers [8–10]. Efforts to disseminate these tools are substantial—they are widely promoted by different organizations (e.g. WFOT, WCPT, ISWP, ISPO), they are translated into several languages, and they are being adopted as the basis for global training [11, 12], and competency evaluations [13].

Despite these dissemination efforts, there has been relatively little change in the wheelchair sector, and governments, private companies, and not-for-profits continue to distribute wheelchairs that would not be considered ‘appropriate’ [6] through the service delivery approach that does not include all 8 steps recommended by WHO [7]. There are two key reasons that organizations do not universally adopt these consensus approaches. First, policies that dictate the type of wheelchair service provision are weak or non-existent in many countries where the need is greatest, and therefore organizations are not obligated to adhere to specific service or product quality standards. Second, there is a paucity of evidence that providing wheelchairs through the approach outlined by WHO addresses the needs for wheelchair users more efficiently or effectively than other approaches.

These two reasons are closely linked and related to a lack of objective evidence about the benefits of providing appropriate wheelchairs through an 8-Steps approach (described by WHO) versus simply giving a standard hospital-style wheelchair to someone who requests it, which continues to be the standard of care in most countries. Subjective evidence indicating that hospital-style wheelchairs fail quickly in the community were published as early as 1990 [4, 14], but investigated only a small number of wheelchairs and were geographically focused on India. Interest in the impact of wheelchair service increased as the sector began to coordinate in 2006 when the WHO became involved [15], and researchers began to collect and publish outcome data. For instance, a cross-sectional study on 188 wheelchair users who received basic wheelchairs without formal service revealed that 93.1% of the wheelchairs were still in use after an average of 18 months and that receiving the wheelchair was associated with a significant increase in independence and significantly decreased pressure ulcer incidence [16]. These strong positive results bolstered the argument that the resource-intensive approach promoted by the WHO may not be necessary. Meanwhile, because the study was cross-sectional and investigated a group who received a single type of wheelchair, it does not provide conclusive evidence of the relative value of providing wheelchairs through WHO’s 8-Steps approach, nor provide reliable insight into whether it was the wheelchair or other factors which led the improvements. The first study we are aware of that investigated the impact of the WHO’s 8-Steps service approach was in Indonesia, and compared a group receiving wheelchairs through the 8-Steps process to a waitlist control group at baseline and a 6-month follow-up [17]. Participants who received new wheelchairs reported significant increases in physical health, environmental health, and satisfaction with their mobility devices as compared to the waitlist control group. Using a longitudinal, mixed-methods study design, this research helps to support WHO’s 8-Steps service provision approach but did not directly compare it to the standard of care. A longitudinal study of 200 individuals who received one of two designs of wheelchairs [18] was conducted in Peru, Uganda, and Vietnam found that overall health indicators, distance traveled, and employment increased, and that wheelchair design had little impact on these results. This study was conducted on a population of users similar to an earlier study [16] and similarly, did not receive services based on the 8-Steps approach, did not include a control group, or use strongly validated outcome measures.

The only study we are aware of that compared service provision models was a cross-sectional study that recorded data from 852 wheelchair users in Kenya and the Philippines [19, 20]. The investigators used a proxy measure for services based on the subject’s self-report of how many service steps (from 0 to 8) occurred when they received their wheelchairs. The results suggest that users in Kenya versus the Philippines were more likely to use their wheelchairs daily (60% vs. 42%) and had higher activities of daily living (ADL) performance (80% vs. 74%) highlighting the country-level differences. The impact of increased services was largely dependent on what service was received. For instance, individuals who were assessed for a wheelchair (Step 2) were more likely to have a higher ADL performance when intervention included being measured or assessed before selecting a wheelchair. Similarly, individuals who received training (Step 7) were more likely to use their wheelchairs daily. This cross-sectional study of a relatively large subject pool provides strong evidence of the positive impact of services on the outcomes of wheelchair service provision.

The prior research evidence paints a positive but incomplete picture of the impact of service provision in the wheelchair sector. As a whole, the studies suggest that wheelchairs have a positive impact on the quality of life and health of wheelchair users, which is consistent with the goals and outcomes in more resourced settings [21], and that the degree to which services are provided increases that impact. However, there is still a significant gap in evidence related to the specific benefits of an 8-Steps service provision approach compared to the standard of care (SOC) of simply distributing standard wheelchairs. The need for this information is becoming increasingly important to meet a global push towards using evidence to drive policy changes related to rehabilitation and assistive health technology purchasing decisions. These goals have been emphasized by global collaborations such as through Call to Action in WHO’s REHAB2030 [22], WHO’s GATE Research Priorities [23], and AT scale [24].

The gaps in previous research along with the global focus on evidence-based decision-making motivated our team to explore wheelchair usage, satisfaction, skills, maintenance and repairs, and life satisfaction among individuals receiving wheelchairs through the SOC and the WHO 8-Steps process. Based on the benefits suggested by the WHO, we anticipated finding increased levels of usage, wheelchair satisfaction, wheelchair skills and reduced repair frequency in the 8-Steps group compared to the SOC group. In addition to gathering evidence of the relative benefits of each service provision model, we also sought to investigate the feasibility and practicality of this type of research in less-resourced settings and collect normative data that can be useful for future studies.

## Methods

A longitudinal, mixed-methods study was carried out to evaluate the impact of wheelchair service provision from three wheelchair providers (WPs) in Indonesia: Puspadi, the Bunga Bali Foundation, and the Social Department. Puspadi is staffed by service providers who were all trained to provide services using the 8-Steps service provision model described in the WHO guidelines [7], whereas Bunga Bali Foundation (BBF) and the Social Department use the standard of care (SOC) to distribute hospital-style wheelchairs to those who requested them without any clinical services.

Three research teams were involved in the project. A team from the Comprehensive Initiative on Technology Evaluation at the Massachusetts Institute of Technology (CITE-MIT) designed the initial study and supported in-country data collection. A team from the Center for Health Policy and Management at Gadjah Mada University (CHPM-GMU) led the data collection efforts in Indonesia. A team from the Department of Rehabilitation Sciences and Technology (RST) from the University of Pittsburgh led data analysis and drafting of this manuscript. The study was supported by a grant from Google.org (grant #322068) which was awarded to United Cerebral Palsy—Wheels for Humanity (UCPW) who contracted the other organizations to carry out the research.

Ethical approval was granted from the Medical and Health Research Ethics Committee (MHREC) of the Faculty of Medicine GMU, the University of Pittsburgh Institutional Review Board (IRB), and MIT Committee on the Use of Human as Experimental Participants (COUHES). Written informed consent was collected for all study participants, minors who participated in the study received written consent from their parents, and assent was given from the research participants. Wheelchair users, defined as a person with mobility limitations requiring a wheelchair as a primary means of mobility, on the waitlist from Puspadi, BBF, and the Social Department were recruited into the study from May to August 2017. Wheelchair users who were 16 years or older, could interact and communicate with the help of their caregiver, were recruited to participate in the study. The target sample size was limited by the size of the waitlist in these organizations, which was just over 200 people, and the number of wheelchairs available.

**Fig 1:**
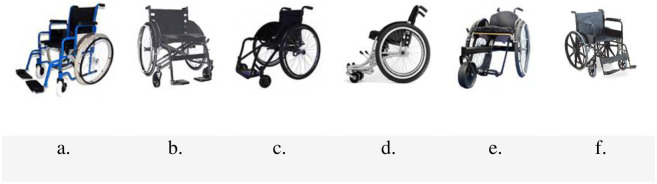
Types of wheelchairs provided. a. TRN, b. AF, c. AR, d. 4AT, e. 3AT, f. H.

Wheelchair users receiving wheelchairs from Puspadi were provided with one of five different wheelchair models according to their needs: Transport (TRN), Active Folding (AF), Active Rigid (AR), 4-wheels All-terrain (4AT), and 3-wheels All-terrain (3AT). These wheelchairs are shown in Fig 1 and described in the S6 Table. Puspadi wheelchair service providers received training to provide wheelchairs according to the WHO 8-Steps approach. Wheelchair users receiving wheelchairs from the SOC group were given a basic hospital-style wheelchair (H) (see Fig 1 and S6 Table).

### Data collection methods and tools

Data was collected from the participants through in-person interviews that were used to record responses to a set of standardized questionnaires. The closed-ended responses were entered into tablets by researchers from the CHPM-GMU team using KoboToolbox, a survey software.

The interview protocol was comprised of questions from a variety of questionnaires including the International Society of Wheelchair Professionals Minimum Uniform Data Set (ISWP-MUD) [25], Wheelchair Skills Test Questionnaire (WST-Q) [26], Life Satisfaction Questionnaire (LiSAT-11) [27], and the Breakdown and Adverse Consequences Questionnaire (BAC-Q) [28]. The questionnaires were adapted in the following way: First, they were all translated into the local Indonesian language, Bahasa, and compared to the English translation. Second, the tools were modified to fit the cultural and linguistic context based on practice interviews with other team members, testing in the field with wheelchair users, and feedback from local partners, such as the United Cerebral Palsy Roda Untuk Kemanusiaan (UCP-RUK), Puspadi, BBF, and CHPM-GMU. The questionnaires were tested with similar types of wheelchair users before the data collection period. Confusing questions were revised or removed, and questions considered culturally inappropriate were eliminated. Finally, the length of the questionnaires was assessed based on the testing, and the questions were prioritized.

Table 1 provides an overview of the types of data collected through each questionnaire. These questionnaires were administered at baseline and endline, which was at least 3 months but not more than 6 months after baseline, to all the wheelchair users who participated in the study.

**Table 1:** Questionnaires administered at baseline and endline (between 3 to 6 months after baseline).

Tool	Data Type
ISWP-MUD	Demographics, self-reported wheelchair usage, satisfaction with wheelchair
WST-Q*	Wheelchair skills
BAC-Q**	Wheelchair maintenance and repairs
LiSAT-11***	Life satisfaction

* Administered to participants who used a manual wheelchair at baseline and endline.

**Administered just at endline.

*** The sexual health item was removed due to the sensitive nature of the question, leaving 10 questions in this tool.

## Data analysis

Baseline demographic characteristics were compared between the 8-Steps group and SOC group using independent samples t-tests and chi-squared tests (for continuous and categorical variables, respectively) to test if groups were comparable.

Descriptive statistics were used to analyze self-reported wheelchair usage data, which is described in terms of days per week, hours per day, distance traveled and the places where the device was used for each group; and the overall change from baseline to endline. The McNemar tests were used to evaluate consistency in the reported settings where wheelchairs were used.

Significant changes in wheelchair skills (as per WST-Q scores) were evaluated for the 8-Steps group. The maximum score that an individual could achieve was 100%. Missing responses were not considered valid scores. Independent samples t-tests were used to determine differences in WST-Q scores at the study endline to differentiate between participants who previously owned wheelchairs and new wheelchair owners. A paired samples t-test within each group was used to compare changes in WST-Q scores between baseline and endline for those participants who owned a manual wheelchair at baseline and used the wheelchair provided during the study. The Wilcoxon signed-ranked test was used to determine differences in WST-Q scores across types of wheelchairs. Wheelchair maintenance and repairs data from the data reported at the endline were analyzed using frequency statistics.

ISWP-MUD gathered information about the participant’s satisfaction with his wheelchair from 1 (not satisfied) to 5 (very satisfied). This information was analyzed using the Wilcoxon-signed rank test to determine changes from baseline to endline. As a secondary exploratory analysis, the Kruskal-Wallis test was used to determine differences in satisfaction for people receiving different wheelchair models.

Life satisfaction (LiSAT-11) an 11-question questionnaire was used to assess global satisfaction across different aspects of life. Study participants completed this questionnaire at the baseline and again at the study endline. Satisfaction levels were self-reported for 10 out of 11 domains, across a six-level scale from 1, very dissatisfied to 6, very satisfied [29]. This questionnaire was analyzed using a paired t-test to determine changes in satisfaction levels from baseline to endline.

## Results

A total of 150 participants were recruited for the study, 15% of whom had not owned wheelchairs previously. A total of eight participants were excluded from data analysis: six that did not participate in the follow-up, and two that were deceased before the conclusion of the study. Therefore, data from 142 participants were analyzed: 118 from the 8-Steps group, and 24 from the SOC group.

Descriptive statistics of age, gender, disability, education level, and mobility aid use are shown for each group in Table 2. No significant differences were found for gender (p = 0.99). Individuals in the SOC group were significantly older (p = .001) and were less likely than the 8-Steps group to report using a mobility aid at enrollment (p = .001; Table 2). There were also differences in reported diagnoses between groups. More than half of the participants recruited in the 8-Steps group had polio (51.7%), whereas no one in the SOC group reported having polio. Due to differences between these two groups, subsequent results are presented separately, and no across-group comparisons were made or implied. Additional analysis of medical diagnoses by type of wheelchair provided is shown in the S1 Table.

**Table 2:** Demographics.

	8-Steps	SOC
	(n = 118)	(n = 24)
	*mean ± SD*	*mean ± SD*
*Age*	40.4 ± 12.6	57.4 ± 15.2
	*n (%)*	*n (%)*
*Female*	36 (30.5)	4 (16.7)
*Owning a mobility aid at baseline*	115 (97.5)	16 (66.6)
*Medical Condition*		
Polio	61 (51.7)	0
Spinal Cord Injury	22 (18.6)	4 (16.7)
Other (unknown)	20 (16.9)	9 (37.5)
Cerebral Palsy	5 (4.2)	0
Muscular Dystrophy	3 (2.5)	1 (4.2)
Osteogenesis Imperfecta	3 (2.5)	1 (4.2)
Amputation	2 (1.7)	0
Brain Injury	1 (0.8)	0
Stroke	1 (0.8)	9 (37.5)
*Education Level*		
None	24 (20.3)	4 (16.7)
Primary	42 (35.6)	14 (58.3)
Secondary	21(17.8)	4 (16.7)
High School +	29 (24.6)	2 (8.3)
*No wheelchair at baseline*	15 (12.7)	16 (66.6)

### Wheelchair usage

Most of the participants from the 8-Steps group used their wheelchair every day (Table 3) and traveled less than 500 meters per day (Table 3) at baseline and endline. Many reported using their wheelchair either 1–3 hours per day or more than 8 hours per day. By observation, the percentage of self-reported daily wheelchair usage was not largely different between baseline and endline (Table 3). For usage measured by ‘days per week,’ the increase in the number of individuals reporting using their wheelchair 1–3 days a week at endline was partially attributed to the individuals who did not have a wheelchair at baseline and to those who stopped using it every day (refer to S2 and S3 Tables describing the change in days per week and distance from baseline to endline of wheelchair usage in this group.) In the SOC group, participants were more likely to use the device every day and travel <100m at both time points (Table 3). 46% of individuals in this group reported using their wheelchair between 1–3 hours per day at endline (Table 3). More detailed information on the change in daily usage per week and the daily distance traveled by this group can be found in S4 and S5 Tables.

**Table 3:** Wheelchair usage descriptive statistics.

		8-Steps (n = 118)		SOC (n = 24)	
		*Baseline*	*Endline*	*Baseline*	*Endline*
		*n (%)*	*n (%)*	*n (%)*	*n (%)*
No WC		15 (12.7)	5 (4.2)	16 (66.7)	3 (12.5)
Days/week	**< 1 day**	3 (2.5)	3 (2.5)	1 (4.2)	0
	**1–3 days**	9 (7.6)	22 (18.6)	0 (0)	6 (25)
	**4–6 days**	3 (2.5)	6 (5.1)	1 (4.2)	3 (12.5)
	**Every day**	88 (74.6)	81 (68.6)	6 (25)	12 (50)
	**Missing**	0	1 (0.8)	0	0
Hrs./day	**< 1 hour**	4 (3.4)	11 (9.3)	2 (8.3)	2 (8.3)
	**1–3 hours**	28 (23.7)	35 (29.7)	0	11 (45.8)
	**4–6 hours**	13 (11)	17 (14.4)	3 (12.5)	4 (16.7)
	**7–8 hours**	4 (3.4)	7 (5.9)	1 (4.2)	0
	**8+ hours**	54 (45.8)	41 (34.7)	2 (8.3)	4 (16.7)
	**Missing**	0	2 (1.7)	0	0
Distance/day	**< 100 m**	40 (33.9)	54 (45.8)	6 (25)	18 (75)
	**100–499 m**	36 (30.5)	31 (26.3)	1 (4.2)	3 (12.5)
	**500–999 m**	16 (13.6)	9 (7.6)	1 (4.2)	0
	**1–5 km**	9 (7.6)	16 (13.6)	0	0
	**5 + km**	2 (1.7)	1 (0.8)	0	0
	**Missing**	0	2 (1.7)	0	0
Places	**School**	10 (8.5)	4 (3.4)^a^	0	0^d^
	**Home**	93 (78.8)	85 (72)^b^	7 (29.2)	21 (87.5)^c^
	**Sports**	21 (17.8)	12 (10.2)^c^	2 (8.3)	0^c^
	**Public- Other**	68 (57.6)	68 (57.6)^c^	3 (12.5)	4 (16.7)^c^
	**Work**	34 (28.8)	20 (16.9)^a^	1 (4.2)	0^c^
	**Transportation**	14 (11.9)	7 (5.9)^c^	1 (4.2)	0^c^
	**Outdoors**	53 (44.9)	31 (26.3)^a^	3 (12.5)	0^c^
	**Leisure**	40 (33.9)	27 (22.9)^a^	3 (12.5)	0^c^

^a^ p < .05.

^b^ p = .001.

^c^ p>.05.

^d^ p-value could not be computed.

The participants from the 8-Steps group reported usage in all settings at both time points (Table 3). The most frequently reported settings were home and other public places, followed by outdoors on rough surfaces and leisure activities. The 8-Steps group had significant negative trends for almost all settings (5 out of 8) (Table 3). For example, 23 individuals at baseline who reported using their wheelchair at ‘home,’ reported not using their wheelchair at ‘home’ at endline. Similar negative changes were seen in other settings like ‘school,’ ‘work,’ ‘outdoors on rough surfaces,’ and ‘leisure activities.’ The only two settings reported by the SOC group at endline were ‘home’ and ‘other public places. The SOC group had very small cell sizes, and thus, the differences were not statistically significant; however, a negative trend in the number of places where the wheelchair was used could also be observed. At the endline, 5(4.2%) participants from the 8-Steps group and 3 (12.5%) individuals from the SOC group reported they were not using the study wheelchair.

When analyzing wheelchair usage by type of wheelchair received, 65% of the individuals receiving any of the wheelchairs reported using it every day, except for individuals who received the 3AT (See S1A Fig). Participants who received a 3AT used their wheelchair either 1–3 days per week or every day. Interestingly, usage in hours per day was bimodal; individuals were most likely to report using their wheelchair 1–3 hours per day or more than 8 hours per day (See S1B Fig). Individuals who received 4AT or TRN chairs were more likely to report using it the most (more than 8 hours per day), while individuals who received H, AR, and 3AT were more likely to report lower usage (1–3 hours per day). Additionally, across wheelchairs most participants (TRN = 42.3%, AF = 60.9%, AR = 40.9%, 4AT = 38.5%, 3AT = 47.6%, H = 75%) reported traveling less than 100 m per day (See S1C Fig). Individuals who received H wheelchairs reported traveling the shortest distance, with no one having this type of wheelchair with a distance traveled over 500 m. Individuals with 4AT and 3ATs seemed to travel the longest distances, although some individuals with TRN, AR, and AF’s did report traveling >1km.

It is important to note that individuals could choose multiple settings where they used their wheelchair. Although wheelchair use at ‘home’ was the most frequently reported setting for all wheelchair types (S1D Fig in WUV file), there were some interesting differences in the other settings by wheelchair type. The participants who used H only reported usage at ‘home’ and ‘other public places. In contrast, those participants who used 4AT tended to report more settings and choose those settings that were less frequently reported by the sample such as ‘sports,’ ‘work,’ and ‘transportation’. AR users also reported usage in all settings but not as high as 4AT users.

### Wheelchair skills

In the 8-Steps group, 15 (12.7%) participants did not own a wheelchair at baseline and did not have baseline WST-Q scores. At endline, the 8-Steps group had 8 (6.9%) participants with non-valid scores. Of the 5 (4.2%) individuals who did not use the wheelchair of the study, 2 (1.7%) also reported not owning a wheelchair at baseline, therefore, the tool was not administered. Thus, the final sample for the 8-Steps group used for comparing baseline and endline scores was 92. Overall, the average WST-Q score decreased from baseline (M = 64.7, SD = 17.9) to endline (M = 58.6, SD = 17.2) with an average decrease of 6.03 points (SD = 10.4). This difference was statistically significant; t (91) = 5.542, p < .001, d = .577. An independent sample t-test was conducted to determine whether there was a difference in WST-Q scores at the endline between those participants who owned a wheelchair at baseline (n = 103) and those who did not own one (n = 15). No significant differences were found.

In the SOC group, 16 participants (66.6%) did not own a wheelchair at baseline and 3 (12.5%) of participants reported never using the study provided wheelchair. Also, one of the participants obtained a zero score at baseline as all the individual skills were responded as “no” when asked about the capacity of performing them. Due to the small sample size, statistical comparisons of WST-Q scores were not performed. The baseline WST-Q mean score (n = 8) was 46.48 (SD = 31.50). Endline WST-Q mean score (n = 21) was 34.31 (SD = 25.29.)

Between 74% and 94% of individuals in the 8-Steps group reported the ability to do basic skills, whereas between 36% and 86% performed intermediate skills (see Table 4). A lesser percentage were able to perform more advanced skills (between 2% and 29%) (also shown in Table 4). Between 42% and 66% of individuals from the SOC group were able to do basic skills, whereas 16% to 58% were able to do intermediate skills. In these groups, some skills were not achieved by any of the individuals.

**Table 4:** Individual skills for WST-Q capacity at endline.

Skill Level	Individual Skill	8-Steps	SOC
		*n (%)*	*n (%)*
Basic	**Rolls forward short distance**	108 (91.5)	12 (50)
Basic	**Rolls backward short distance**	103 (87.3)	13 (54.2)
Basic	**Turns in place**	103 (87.3)	15 (62.5)
Basic	**Turns while moving forward**	111 (94.1)	16 (66.7)
Basic	**Turns while moving backward**	106 (89.8)	13 (54.2)
Basic	**Maneuvers sideways**	105 (89)	10 (41.7)
Basic	**Reaches high object**	64 (54.2)	6 (25)
Basic	**Pick object from floor**	96 (81.4)	13 (54.2)
Basic	**Operate body positioning options**	88 (74.6)	12 (50)
Basic	**Relives weight from buttocks**	99 (83.9)	13 (54.2)
Basic	**Level transfer**	101 (85.6)	14 (54.2)
Intermediate	**Folds and unfolds wheelchair**	71 (60.2)	7 (29.2)
Intermediate	**Gets through hinged door**	90 (76.3)	8 (33.3)
Intermediate	**Rolls longer distance**	87 (73.7)	12 (50)
Intermediate	**Avoids moving obstacles**	91 (77.1)	8 (33.3)
Intermediate	**Ascends slight incline**	89 (75.4)	5 (20.8)
Intermediate	**Descends slight incline**	98 (81.3)	8 (33.3)
Intermediate	**Rolls across side-slope**	64 (54.2)	7 (29.2)
Intermediate	**Rolls on soft surface**	102 (86.4)	14 (58.3)
Intermediate	**Gets over threshold**	89 (75.4)	7 (29.2)
Intermediate	**Gets over gap**	43 (36.4)	5 (20.8)
Intermediate	**Ascends low curb**	46 (39)	4 (16.7)
Intermediate	**Descends low curb**	53 (44.9)	8 (33.3)
Advanced	**Ascends steep incline**	18 (15.3)	0
Advanced	**Descends steep incline**	35 (29.7)	0
Advanced	**Ascends high curb**	11 (9.3)	1 (4.2)
Advanced	**Descend high curb**	24 (20.3)	2 (8.3)
Advanced	**Performs stationary wheelie**	29 (24.6)	3 (12.5)
Advanced	**Turns in place in wheelie position**	19 (16.1)	2 (8.3)
Advanced	**Descends high curb in wheelie position**	11 (9.3)	0
Advanced	**Descends steep incline in wheelie position**	13 (11)	0
Advanced	**Gets from the ground into wheelchair**	79 (66.9)	10 (41.7)
Advanced	**Ascends stairs**	8 (6.8)	2 (8.3)
Advanced	**Descends stairs**	3 (2.5)	0

**Fig 2:**
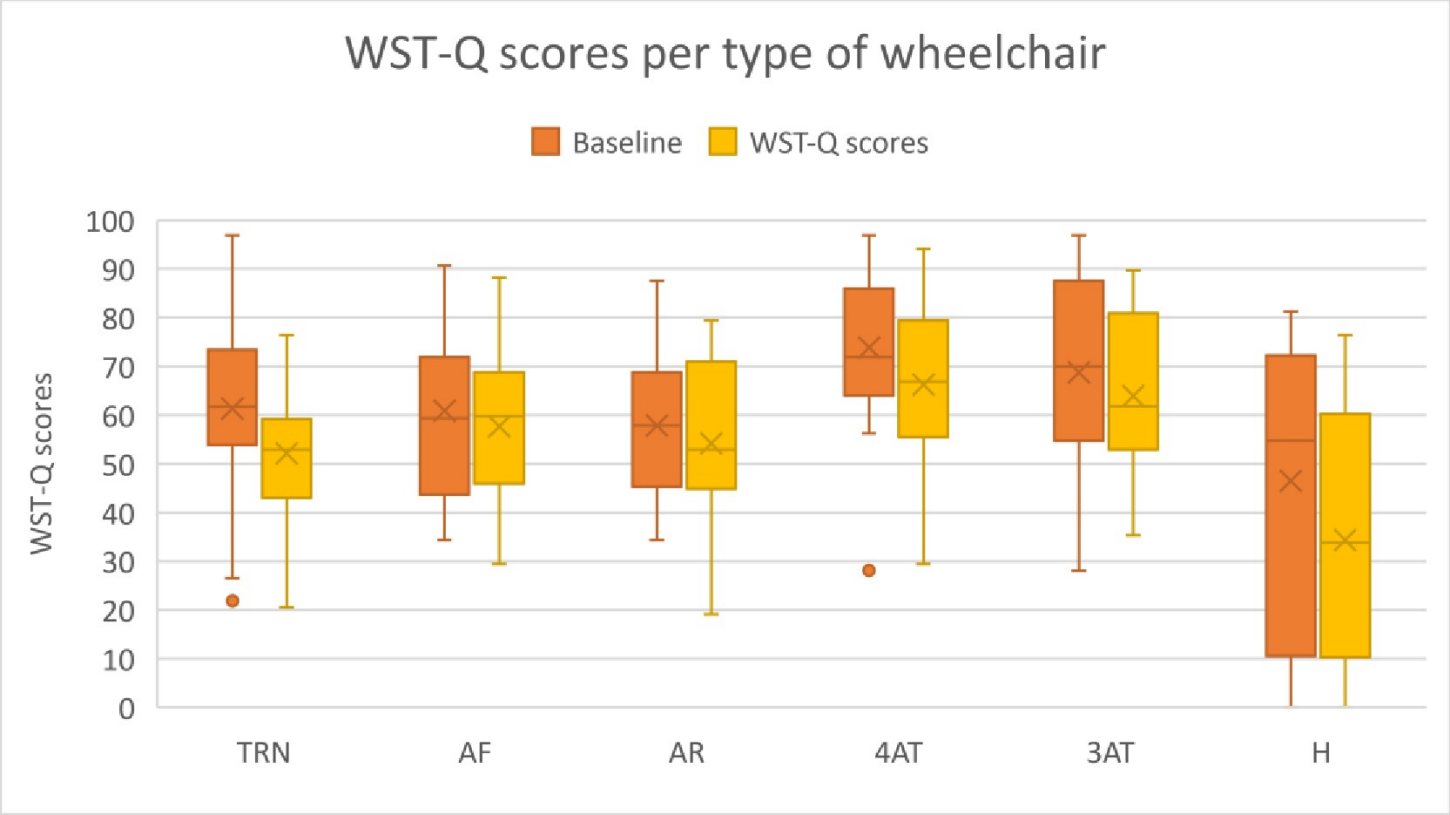
WST-Q scores per type of wheelchair.

Analysis of WST-Q total scores by wheelchair type found that 4AT and 3AT users reported the highest average wheelchair skills scores (66% and 64%), followed by AF users (58%), and AR and TRN users (54% and 52). H users scored the lowest scores (34%). (Fig 2). However, the Wilcoxon signed-rank test showed that there was a significant decrease in WST-Q scores at the end of the study only for TRN users (Z = -3.46, p<0.001) but no significant differences were found for the other types of wheelchairs. No statistically significant results were found for medical diagnosis based on WST-Q scores at baseline or endline.

### Device satisfaction

Wheelchair satisfaction in the 8-Steps group had a rating of M = 4.06, SD = 1.04 at baseline and M = 4.15, SD = .99 at endline. Satisfaction levels ranged from 1 (not satisfied) to 5 (very satisfied). The SOC group was slightly less satisfied overall at baseline (M = 3.88, SD = .83), but presented an increase (M = 4.28, SD = .64) at endline. However, no significant differences were found for the participants’ satisfaction rate about the device between both time points. Table 5 shows the satisfaction rate per type of wheelchair. The missing column corresponds to those users who chose not to respond to the question, and those who reported not having used the wheelchair from the study were not administered this question. No significant differences were found when compared across wheelchairs.

**Table 5:** Satisfaction rate based on the type of wheelchair.

	Not satisfied				Very Satisfied	
	*1*	*2*	*3*	*4*	*5*	*Missing*
	*n (%)*	*n (%)*	*n (%)*	*n (%)*	*n (%)*	*n (%)*
	1 (3.8)	1 (3.8)	2 (7.7)	7 (26.9)	12 (46.2)	2 (7.7)
TRN	0 (0)	2 (8.7)	2 (8.7)	9 (39.1)	8 (34.8)	0 (0)
AF	0 (0)	2 (9.1)	2 (9.1)	9 (40.9)	9 (40.9)	0 (0)
AR	0 (0)	1 (3.8)	3 (11.5)	7 (26.9)	14 (53.8)	1 (3.8)
4AT	0 (0)	0 (0)	5 (23.8)	6 (28.6)	7 (33.3)	1 (4.8)
3AT	0 (0)	0 (0)	2 (8.3)	11 (45.8)	8 (33.3)	0 (0)
H	1 (3.8)	1 (3.8)	2 (7.7)	7 (26.9)	12 (46.2)	2 (7.7)

1 = not satisfied to 5 = very satisfied.

### Wheelchair maintenance and repair

34 participants (28.8%) in the 8-Steps group reported having wheelchairs that stopped functioning correctly or that broke at 3 to 6 months. The most common breakdown reported was one- or two-wheel locks no longer function properly (n = 9, 7.6%), followed by a bearing that stopped turning smoothly (n = 8, 6.8%). Some other wheelchair repairs included tire replacement and broken wheels. Of those repairs recorded, 12 (10.2%) were performed by the participant or a family member followed by the service that provided the wheelchair (n = 11, 9.3%). Two individuals (8%) in the SOC group had wheelchairs that stopped functioning correctly or had a broken wheel. In both instances, the participant or a family member performed the repair.

In the 8-Steps group, 72.7% of participants reported performing wheelchair maintenance over 6-months. The most-reported maintenance activity was wiping or washing the wheelchair (45.8%) followed by adding oil (16.9%) and adding air to the tires (9.3%). Participants or family members did most of the wheelchair repairs (66.9%). A total of 9 (37.5%) participants in the SOC group reported performing maintenance activities, 4 (16.7%) participants reported wiping or washing the wheelchair followed by 2 (8.3%) who added air to the tires. All participants that reported wheelchair maintenance, mentioned it was performed by the participant or a family member.

### Life satisfaction

The 8-steps group reported increased mean satisfaction in life as a whole from baseline (3.9 ± 1.4) to endline (4.2 ± 1.1), p = 0.020. as shown in Table 6. The 8-steps group also reported greater mean satisfaction levels at the study endline for life as a whole (p = .020), contact with friends (p = .049), family life (p = .002), and psychological health (p = .014). The SOC group reported greater mean satisfaction levels for economy (p = 0.009) and psychological health (p = .022) at the study endline.

**Table 6:** LiSAT-11 8-steps and SOC groups self-reported satisfaction at baseline and endline.

	*8-Steps (n = 118)*			*SOC (n = 24)*		
	*Baseline*	*Endline*	*p*	*Baseline*	*Endline*	*p*
	*Mean* ± (*SD*)	*Mean* ± (*SD*)		*Mean* ± (*SD*)	*Mean* ± (*SD*)	
Life as a Whole	3.9 (1.4)	4.2 (1.1)	.020	3.7 (1.3)	4.1 (1.2)	.274
Vocation	4.0 (1.4)	4.3 (1.2)	.089	3.3 (1.6)	3.2 (1.5)	.870
Economy	3.4 (1.4)	3.5 (1.4)	.413	3.6 (1.3)	2.7 (1.3)	.009
Leisure	3.9 (1.4)	4.2 (1.2)	.053	4.4 (1.0)	4.8 (0.4)	.163
Contact	4.8 (0.5)	4.7 (0.8)	.049	4.1 (1.4)	4.7 (0.9)	.254
ADLs	4.7 (0.9)	4.8 (0.9)	.413	4.1 (1.3)	4.4 (1.1)	.230
Family Life *	4.4 (1.1)	4.8 (1.0)	.002	4.6 (0.9)	4.3 (1.2)	.246
Partner Relationship **	4.8 (0.8)	4.6 (1.3)	.247	4.7 (0.8)	4.6 (1.1)	.719
Physical Health	4.2 (1.3)	4.2 (1.2)	.951	4.1 (1.3)	3.9 (1.5)	.634
Psychological Health	4.2 (1.2)	4.5 (1.1)	.014	4.6 (1.0)	3.9 (1.5)	.022

*participants living with ≥ 1 family member, 8-Steps: n = 110, SOC: n = 23.

**participants who reported having a partner, 8-Steps: n = 55, SOC: n = 16.

## Discussion

This study describes the characteristics of wheelchair usage, satisfaction, skills, maintenance and repairs, and life satisfaction of individuals who received wheelchairs following either the WHO 8-Steps or the SOC in Indonesia. Due to the significant differences in age and medical diagnosis, across-group statistical comparisons were not appropriate. Given the limited data on the outcomes of wheelchair provision globally, our data contributes to the body of knowledge on the impact of different wheelchair products and delivery processes and provides normative data that can be used for future studies.

### Wheelchair usage

Although participants in the 8-Steps group reported using their wheelchairs every day, they did not report traveling long distances relative to other populations of wheelchair users and 40% use their wheelchair for less than 3 hours per day. It has been documented that wheelchair users in more-resourced settings travel longer distances (>2km) and use their wheelchairs more (>8 hours per day) [30]. Low mobility levels from wheelchair users in this study may suggest that they may be facing additional personal, sociopolitical, and environmental barriers to personal mobility such as decreased health, limited ability to maneuver the wheelchair, inappropriate match between the product and the client’s needs, lack of accessible environments, etc. [30, 31].

Wheelchair usage in the SOC group was also relatively low. SOC group used their wheelchairs every day for very short distances (<100m), short times (<3 hours), and few places (home and public places). Considering that at least 62.5% of users in this group had a known permanent disabling condition, and no wheelchair assessment was performed on any of them, these devices may not have met the physical, environmental, and mobility needs of the users and which may have been reflected in their low mobility levels. Training wheelchair providers about how to assess, select and fit a wheelchair, as well as, increasing the variety of wheelchair modes available is recommended to increase wheelchair usage among people with mobility limitations.

### Wheelchair skills

Total scores for wheelchair skills in this study were low for all participants (58.6% and 34.3%) relative to the skills reported in the literature of over cohorts (84% [31] and 88% [32]). Reasons for these differences may be attributed to study participants’ health status, matching between product and user’s needs, and the dose of wheelchair skills training and practice for the user. In this study, the wheelchair skills program taught to the 8-steps group only included seven basic skills (pushing, turning, going up/down slopes, going up/down steps with assistance, and performing a partial wheelie), it did not include intermediate or advanced skills.

Therefore, an insufficient amount of training and practice were provided to ensure skill transfer as all the 32 wheelchair skills included in the WST-Q cannot be learned in a few hours. Intermediate and advanced skills are necessary for mobility in different environments indoors and outdoors as not being able to perform them can affect independence, community participation, self-esteem, work, and school attendance, and more [17]. More wheelchair skills education and supervised practice could be beneficial to wheelchair users and service providers. This should be considered not only in the WHO WSTP packages but also in educational programs as mentioned by Fung et al. [33].

Other factors that could have affected wheelchair skills are the characteristics of the different wheelchair models provided and how well these were matched and adjusted to the user’s physical, functional, and environmental needs. For instance, less active users, who have decreased function and strength, and require a wheelchair only for indoor mobility should not be provided with a heavy or sturdy wheelchair intended for rough terrain mobility as the weight and overall length may interfere with their ability to maneuver it in tight spaces. Active users, with more function, strength, and body control, should get a more adjustable, modular, and configurable wheelchair that meets their physical dimensions and environmental needs, either urban or rural. [19, 31]. The study personnel were trained using the WHO 8-steps for appropriate wheelchair provisions, but there was no assessment to evaluate whether all 8-steps were being applied as directed by the WHO. Therefore, it is possible that the 8-steps model was not always adhered to exactly, which is a limitation of this study. Wheelchair provisions is an emerging profession in low- and middle-income counties, a standard of certification and training for wheelchair prescription has not yet been established. Wheelchair prescription requires unique skills and experience and may be sparsely included in pre-professional health science programs. This highlights the increased need for standardized training for wheelchair provisions. [34].

### Wheelchair maintenance and repair

After 3 to 6 months of wheelchair use, about one-third of 8-Steps users and on- tenth of SOC users performed minor repairs to their wheelchairs. The SOC group did not receive any training on wheelchair maintenance and repair. The 8-Steps group did receive training on wheelchair maintenance and repairs. Training in wheelchair maintenance increases the frequency that wheelchair users perform these activities to their wheelchairs, which may, in turn, keep the device in good working condition, increase the life span of their device, and decrease the adverse effects of wheelchair failure [35].

### Device and life satisfaction

Wheelchair satisfaction was high and remained the same for previous and new devices and across the 6 wheelchair types. The grateful nature of the Indonesian culture may be related to this finding. Future research should investigate the factors that influence satisfaction with wheelchairs provided in LMIC such as the weight, design, ease of use, adjustability, durability, etc. while compensating for cultural elements that may bias responses. Study participants for both groups indicated increased levels of satisfaction for life as a whole as indicated by the LiSAT-11 questionnaire. The 8-Steps group participants increased mean satisfaction levels in every domain except for partner relationship, which was only reported for a smaller sample of individuals, (n = 55) who indicated they had a partner. The SOC group reported less satisfaction in vocation and economy domains. These results are as expected considering the low employment rates and economic participation of people with disabilities globally.

## Limitations

The most significant limitation of the study was that the subject groups were not randomly assigned to wheelchair groups. Individuals from the 8-Steps group and the SOC group were recruited from organizations with ongoing wheelchair services that provided care to different populations that made across-group comparisons impossible. Ethically, random assignments of 8-Steps and SOC may not be appropriate given that the 8-Steps are globally recognized best-practices. Therefore, future studies should be either use a wait-list control group or perform subject matching across organizations providing two different service approaches (i.e., 8-Steps vs SOC).

Although service providers at Puspadi were formally trained in the 8-Steps process, it was noted by the research team that at times there were not enough resources to fully complete the 8-Steps process, and therefore services may not have met the WHO standards. In addition, some of the wheelchair users received a wheelchair three to four months before the baseline. This created challenges with recall bias and confusion about which wheelchair type the data collectors were referring to in the study questions. This fact and the limited study timeline of 3 to 6 months may have biased our results, as the impact of wheelchair services may not be realized until later.

Another limitation of this study was the difficulty of collecting data from participants from both groups at baseline and endline interviews. Even though all questionnaires were translated into local languages and responses were translated into English, the reliability of the translated questionnaires is still unknown, questionnaires were not back translated to English due to limited time.

Caution should be taken when interpreting the results as they may be highly influenced by the status in health, age, and other sociodemographic indicators of the participants.

## Conclusion

Our results indicated that the training performed did not successfully impact wheelchair skills in the long term. Training may have to be adapted for dose, population, and other mitigating reasons to determine why skills were reduced over time. There is a need for wheelchair users to learn how to effectively use and maintain their wheelchairs and translate this knowledge to improve satisfaction and quality of life. Our study highlights many of the challenges of performing outcomes research in this population and environment that should be taken into consideration when designing robust research studies in less-resourced environments. More rigorous studies that evaluate the impact of the WHO 8-Steps method for wheelchair service provision should consider the quality of services provided.

## Supporting information

S1 Table Number of study participants divided by diagnosis and type of wheelchair provided. (DOCX)

S2 Table Number of participants using their wheelchair before and after wheelchair service provision in the 8-Steps group [days per week]. This table shows representative data for wheelchair usage before and after the provision of associated services and products. The number of participants who demonstrate a change in wheelchair usage following the WHO 8-Steps are reflected in unshaded cells.
(DOCX)

S3 Table Number of participants using their wheelchair before and after wheelchair service provision in the 8-Steps group [distance per day]. This table shows representative data for wheelchair usage before and after the provision of associated services and products. The number of participants who demonstrate a change in wheelchair usage following the WHO 8-Steps are reflected in unshaded cells.
(DOCX)

S4 Table Number of participants using their wheelchair before and after wheelchair service provision in the SOC group [days per week]. This table shows representative data for wheelchair usage before and after the provision of associated services and products. The number of participants who demonstrate a change in wheelchair usage following the WHO 8-Steps are reflected in unshaded cells.
(DOCX)

S5 Table Number of participants using their wheelchair before and after wheelchair distribution in the SOC group [distance per day]. This table shows representative data for wheelchair usage before and after the delivery of a wheelchair without associated services. The number of participants who demonstrate a change in wheelchair usage following the wheelchair distribution is reflected in unshaded cells.
(DOCX)

S6 Table Detailed description of the study wheelchairs. (DOCX)

S1 Fig Wheelchair usage per type of wheelchair. Stacked columns graphs to compare number of participants using their wheelchairs across wheelchair types in days per week, hours per day, distance traveled, and places visited. S1A Fig. Wheelchair usage in days per week by type of wheelchair. S1B Fig. Wheelchair usage in hours per day by type of wheelchair. S1C Fig. Distance traveled per day by type of wheelchair. S1D Fig. Places visited by type of wheelchair.
(DOCX)
