# Supplementary material for: Exploratory investigation of the outcomes of wheelchair provision through two service models in Indonesia
Source: PLoS One. 2021 Jun 1;16(6):e0228428. doi: 10.1371/journal.pone.0228428 (PMC8168880; doi:10.1371/journal.pone.0228428)
Supplement: S1 Table — (DOCX) [file pone.0228428.s001.docx]

# S1. Table. Number of study participants divided by diagnosis and type of wheelchair provided.

| \| ***Diagnosis*** \| **Type of wheelchair** \| \| \| \| \| \| \| --- \| --- \| --- \| --- \| --- \| --- \| --- \| \| Disability \| TRN \| AF \| AR \| 4AT \| 3AT \| H \| \| Polio \| 14 \| 13 \| 8 \| 13 \| 13 \| 0 \| \| Spinal Cord Injury \| 4 \| 5 \| 3 \| 6 \| 4 \| 4 \| \| Other (unknown) \| 4 \| 3 \| 5 \| 6 \| 2 \| 9 \| \| Cerebral Palsy \| 0 \| 0 \| 4 \| 1 \| 0 \| 0 \| \| Muscular Dystrophy \| 2 \| 0 \| 1 \| 0 \| 0 \| 1 \| \| Osteo. Imperfecta \| 0 \| 1 \| 0 \| 0 \| 2 \| 1 \| \| Amputation \| 1 \| 1 \| 0 \| 0 \| 0 \| 0 \| \| Brain Injury \| 0 \| 0 \| 1 \| 0 \| 0 \| 0 \| \| Stroke \| 1 \| 0 \| 0 \| 0 \| 0 \| 9 \| \| Total \| 26 \| 23 \| 22 \| 26 \| 21 \| 24 \| |  |
| --- | --- | --- | --- | --- | --- | --- | --- | --- | --- | --- | --- | --- | --- | --- | --- | --- | --- | --- | --- | --- | --- | --- | --- | --- | --- | --- | --- | --- | --- | --- | --- | --- | --- | --- | --- | --- | --- | --- | --- | --- | --- | --- | --- | --- | --- | --- | --- | --- | --- | --- | --- | --- | --- | --- | --- | --- | --- | --- | --- | --- | --- | --- | --- | --- | --- | --- | --- | --- | --- | --- | --- | --- | --- | --- | --- | --- | --- | --- | --- | --- | --- | --- | --- | --- | --- |
