# Supplementary material for: Exploratory investigation of the outcomes of wheelchair provision through two service models in Indonesia
Source: PLoS One. 2021 Jun 1;16(6):e0228428. doi: 10.1371/journal.pone.0228428 (PMC8168880; doi:10.1371/journal.pone.0228428)
Supplement: S2 Table — This table shows representative data for wheelchair usage before and after the provision of associated services and products. The number of participants who demonstrate a change in wheelchair usage following the WHO 8-Steps are reflected in unshaded cells. (DOCX) [file pone.0228428.s002.docx]

# S2. Table. Number of subjects using their wheelchair before and after wheelchair service provision in the 8-Steps group [days per week]. This table shows representative data for wheelchair usage before and after the provision of associated services and products. The number of subjects who demonstrate a change in wheelchair usage following the WHO 8-Steps are reflected in unshaded cells.

| Baseline | Endline | | | | | | | |
| --- | --- | --- | --- | --- | --- | --- | --- | --- |
|  |  | No WC | < 1 day | 1-3 days | 4-6 days | Everyday | Missing | **Total** |
|  | No WC | 2 | 0 | 5 | 1 | 7 | 0 | 15 |
|  | < 1 day | 0 | 0 | 3 | 0 | 0 | 0 | 3 |
|  | 1-3 days | 1 | 2 | 5 | 1 | 0 | 0 | 9 |
|  | 4-6 days | 0 | 0 | 1 | 1 | 1 | 0 | 3 |
|  | Everyday | 2 | 1 | 8 | 3 | 73 | 1 | 88 |
|  | **Total** | 5 | 3 | 22 | 6 | 81 | 1 | 118 |
