# Supplementary material for: Exploratory investigation of the outcomes of wheelchair provision through two service models in Indonesia
Source: PLoS One. 2021 Jun 1;16(6):e0228428. doi: 10.1371/journal.pone.0228428 (PMC8168880; doi:10.1371/journal.pone.0228428)
Supplement: S3 Table — This table shows representative data for wheelchair usage before and after the provision of associated services and products. The number of participants who demonstrate a change in wheelchair usage following the WHO 8-Steps are reflected in unshaded cells. (DOCX) [file pone.0228428.s003.docx]

# S3. Table. Number of subjects using their wheelchair before and after wheelchair service provision in the 8-Steps group [distance per day] This table shows representative data for wheelchair usage before and after the provision of associated services and products. The number of subjects who demonstrate a change in wheelchair usage following the WHO 8-Steps are reflected in unshaded cells.

| Baseline | Endline | | | | | | | | | |
| --- | --- | --- | --- | --- | --- | --- | --- | --- | --- | --- |
|  |  | No WC | < 100 m | 100 m - 499 m | 500 m - 999 m | | 1-5 km | > 5 km | Missing | **Total** |
|  | No WC | 2 | 8 | 2 | 2 | 1 | | 0 | 0 | 15 |
|  | < 100 m | 1 | 25 | 8 | 2 | 2 | | 0 | 2 | 40 |
|  | 100 m - 499 m | 2 | 16 | 11 | 4 | 3 | | 0 | 0 | 36 |
|  | 500 m - 999 m | 0 | 4 | 4 | 0 | 7 | | 1 | 0 | 16 |
|  | 1-5 km | 0 | 1 | 4 | 1 | 3 | | 0 | 0 | 9 |
|  | > 5 km | 0 | 0 | 2 | 0 | 0 | | 0 | 0 | 2 |
|  | Total | 5 | 54 | 31 | 9 | 16 | | 1 | 2 | 118 |
