# Supplementary material for: Exploratory investigation of the outcomes of wheelchair provision through two service models in Indonesia
Source: PLoS One. 2021 Jun 1;16(6):e0228428. doi: 10.1371/journal.pone.0228428 (PMC8168880; doi:10.1371/journal.pone.0228428)
Supplement: S4 Table — This table shows representative data for wheelchair usage before and after the provision of associated services and products. The number of participants who demonstrate a change in wheelchair usage following the WHO 8-Steps are reflected in unshaded cells. (DOCX) [file pone.0228428.s004.docx]

# S4. Table. Number of subjects using their wheelchair before and after wheelchair service provision in the SOC group [days per week]. This table shows representative data for wheelchair usage before and after the provision of associated services and products. The number of subjects who demonstrate a change in wheelchair usage following the WHO 8-Steps are reflected in unshaded cells.

| Baseline | Endline | | | | | | |
| --- | --- | --- | --- | --- | --- | --- | --- |
|  |  | No WC | < 1 day | 1-3 days | 4-6 days | Everyday | **Total** |
|  | No WC | 3 | 0 | 5 | 2 | 6 | 16 |
|  | < 1 day | 0 | 0 | 1 | 0 | 0 | 1 |
|  | 1-3 days | 0 | 0 | 0 | 0 | 0 | 0 |
|  | 4-6 days | 0 | 0 | 0 | 1 | 0 | 1 |
|  | Everyday | 0 | 0 | 0 | 0 | 6 | 6 |
|  | **Total** | 3 | 0 | 6 | 3 | 12 | 24 |
