# Supplementary material for: Exploratory investigation of the outcomes of wheelchair provision through two service models in Indonesia
Source: PLoS One. 2021 Jun 1;16(6):e0228428. doi: 10.1371/journal.pone.0228428 (PMC8168880; doi:10.1371/journal.pone.0228428)
Supplement: S5 Table — This table shows representative data for wheelchair usage before and after the delivery of a wheelchair without associated services. The number of participants who demonstrate a change in wheelchair usage following the wheelchair distribution is reflected in unshaded cells. (DOCX) [file pone.0228428.s005.docx]

# S5. Table. Number of subjects using their wheelchair before and after wheelchair distribution in the SOC group [distance per day]. This table shows representative data for wheelchair usage before and after the delivery of a wheelchair without associated services. The number of subjects who demonstrate a change in wheelchair usage following the wheelchair distribution is reflected in unshaded cells.

| Baseline | Endline | | | | | | | |
| --- | --- | --- | --- | --- | --- | --- | --- | --- |
|  |  | No WC | < 100 m | 100 m- 499 m | 500 m - 999 m | 1-5 km | > 5 km | **Total** |
|  | No WC | 3 | 12 | 1 | 0 | 0 | 0 | 16 |
|  | < 100 m | 0 | 6 | 0 | 0 | 0 | 0 | 6 |
|  | 100 m - 499 m | 0 | 0 | 1 | 0 | 0 | 0 | 1 |
|  | 500 m - 999 m | 0 | 0 | 1 | 0 | 0 | 0 | 1 |
|  | 1-5 km | 0 | 0 | 0 | 0 | 0 | 0 | 0 |
|  | > 5 km | 0 | 0 | 0 | 0 | 0 | 0 | 0 |
|  | **Total** | 3 | 18 | 3 | 0 | 0 | 0 | 24 |
