# Supplementary material for: Exploratory investigation of the outcomes of wheelchair provision through two service models in Indonesia
Source: PLoS One. 2021 Jun 1;16(6):e0228428. doi: 10.1371/journal.pone.0228428 (PMC8168880; doi:10.1371/journal.pone.0228428)
Supplement: S6 Table — (DOCX) [file pone.0228428.s006.docx]

#####

# S6. Table. Detailed description of the study wheelchairs.

| **Wheelchair Model** | **Manufacturer** | **Description** |
| --- | --- | --- |
| **T**ransport **(TRN)** | **INTCO** | Classic folding X-Brace frame wheelchair. 18” seat depth and different seat widths from 12” to 20”. 18” backrest height (not adjustable.) Upholstery seat and backrest. Seat angle at 4°. Fixed seat height. Flip-up armrests, and height-adjustable removable and swing-away footrests. 24” solid rear wheels with pull-to-lock wheel locks. 8” x 2” polyurethane front casters. Not adjustable center of gravity. Includes polyurethane foam comfort contoured cushion with stretchable waterproof fabric cover. Weight capacity 100kg. Weight: 20kg. Overall length: 37” – 41”. ISO 7176-6. CE mark. ISO 9001. |
| **Intended use:**  Temporary users, including adults without postural support needs, less active users, and older adults (over 65 years old) who do not use the chair more than 3 hours per day.  Not suitable for users who require postural support or who go outside frequently.  Not suitable for users who self-propel significant distances.  Intended for indoors as a transport wheelchair. | |  |
| **Active Folding (AF)** | **Motivation Charitable Trust** | Folding X-Brace frame wheelchair. Adjustable seat depth and different seat widths from 14”to 19”. Padded upholstery seat and backrest, both with tension-adjustable straps. Not adjustable seat height. Height-adjustable backrest. Fixed curved armrest that serve as cloth guards. Height- and angle-adjustable flip-up footrest. 26” pneumatic rear wheels with adjustable pull-to-lock wheel locks, and 6” x 2” solid rubber front casters. Two center of gravity positions. Includes pressure relieving cushion (contoured molded polyurethane foam) with cover. Weight capacity 113kg. Weight: 23kg. Overall length: 38”. ISO 7176-6. CE mark. ISO 9001. ISO 13845. |
| **Intended use:**  Active adults, including older adults (over 65 years old), and older children/teenagers without postural support needs who use the wheelchair as their primary method of mobility and are able to self-propel.  Intended for use indoors or outdoors.  Easy to transport as frame folds. | |  |
| **Active Rigid (AR)** | **UCP Wheels for Humanity** | Rigid frame wheelchair. Adjustable seat depth and different seat widths from 12 3/8” to 18 ½”. Upholstery seat and upholstery backrest with tension adjustable straps. Height-adjustable seat with a 4° angle. Angle- and height- adjustable fold-down backrest. Height-adjustable footplate and curved removable armrests that serve as cloth guards. 24” pneumatic rear wheel with quick-release axles, 4° of camber, and push-to-lock wheel locks. 5” x 1 ½” front casters. Small wheelbase for easy maneuverability in tight spaces. Adjustable center of gravity. Includes polyurethane foam comfort contoured cushion with waterproof fabric cover. Weight capacity 100-130kg. Weight: 15.6-17kg. Overall length: 34”- 38”. CE Mark, ISO 9000, ISO 7176. |
| **Intended use:**  Active children and adults, including older adults (over 65 years old) without postural support needs who use the wheelchair as their primary method of mobility and are able to self-propel.  Intended for use indoors or outdoors in urban settings.  Easy to transport as backrest folds down and rear wheels are removable. Highly adjustable. | |  |
| **4-Wheel All terrain (4AT)** | **Whirlwind International** | Folding X-Brace frame wheelchair. Adjustable seat depth and different seat widths from 12 ½” to 18 ½”. Upholstery seat and backrest with tension adjustable straps. 12° seat angle. Height-adjustable backrest. Height-adjustable footrests and low-profile fixed armrests that serve as cloth guards. 24” pneumatic rear wheel with fixed axles, 3° of camber, and pull-to-lock wheel locks. 4” x 3” front casters. Long wheelbase for stability in uneven terrain. Adjustable center of gravity. Includes pressure relief polyurethane foam cushion with waterproof fabric cover. Weight capacity 100kg. Weight: 20kg. Overall length: 36” – 39”. ISO 7176, FDA, CE Mark, ISO 13485. |
| **Intended use:**  Active adult users, including older adults (over 65 years old), teenagers, and older children without postural support needs who use the wheelchair as their primary method of mobility and are able to self-propel.  Intended for use outdoors and rough terrains. | |  |
| **3-Wheel All Terrain (3AT)** | **Motivation Charitable Trust** | 3-wheel rigid frame wheelchair. Adjustable seat depth and different seat widths from 14” to 19”. Padded upholstery seat and backrest. Height- and angle-adjustable fold-down backrest. 26” pneumatic rear wheels with pull-to-lock wheel locks and quick-release axles. 9” x 2” front rubber casters. Height-adjustable footrests. Removable armrests. Long wheelbase for safe and stable mobility over uneven ground. Adjustable center of gravity. Includes pressure relief polyurethane foam cushion with water-resistant fabric cover. Weight capacity: 113kg. Weight: 22 kg. Overall length: 48”. ISO 7176, CE Mark, ISO 13485, ISO 14001. |
| **Intended use:**  Active adult users, older adults (over 65 years old), and teenagers/older children who use the wheelchair as their primary method of mobility, are able to self-propel, and do not require additional postural support.  Intended for use outdoors and long-distance mobility; suitable for use in rugged conditions and on uneven surfaces.  Not easy to transport. | |  |
| **Hospital-Style (H)** |  | Classic folding X-Brace frame wheelchair. One standard size seat and backrest made of vinyl upholstery. Fixed seat depth and width, fixed backrest height and angle. 24” rear wheels with fixed axles and pull-to-lock wheel locks. 8” diameter front casters. Fixed footrests. Fixed armrests. Fixed center of gravity. No seat cushion included. Weight: 20kg. CE Mark. |
| **Intended use:**  Persons who require a temporal mobility aid to move indoors for very short distance and time.  Institutional mobility mainly. Not appropriate for daily personal mobility or for long time use. | |  |
