# Supplementary material for: Exploratory investigation of the outcomes of wheelchair provision through two service models in Indonesia
Source: PLoS One. 2021 Jun 1;16(6):e0228428. doi: 10.1371/journal.pone.0228428 (PMC8168880; doi:10.1371/journal.pone.0228428)
Supplement: S1 Fig — Stacked columns graphs to compare number of participants using their wheelchairs across wheelchair types in days per week, hours per day, distance traveled, and places visited. S1A Fig. Wheelchair usage in days per week by type of wheelchair. S1B Fig. Wheelchair usage in hours per day by type of wheelchair. S1C Fig. Distance traveled per day by type of wheelchair. S1D Fig. Places visited by type of wheelchair. (DOCX) [file pone.0228428.s007.docx]

| S1. Fig. Wheelchair usage per type of wheelchair. Staked columns graphs to compare number of subjects using their wheelchairs across wheelchair types in days per week, hours per day, distance traveled, and places visited. **S1A. Fig. Wheelchair usage in days per week by type of wheelchair**  **S1B. Fig. Wheelchair usage in hours per day by type of wheelchair**  **S1C. Fig. Distance traveled per day by type of wheelchair**  **S1D. Fig. Places visited by type of wheelchair** |
| --- |
